# Redox Regulation of the Tumor Suppressor PTEN by Hydrogen Peroxide and *Tert*-Butyl Hydroperoxide

**DOI:** 10.3390/ijms18050982

**Published:** 2017-05-10

**Authors:** Ying Zhang, Seong-Jeong Han, Iha Park, Inyoung Kim, Kee-Oh Chay, Seok Mo Kim, Dong Il Jang, Tae-Hoon Lee, Seung-Rock Lee

**Affiliations:** 1 Department of Biochemistry, Chonnam National University Medical School, Gwangju 501-190, Korea; 166560@live.jnu.ac.kr (Y.Z.); sjhan@cotde.co.kr (S.-J.H.); ip071@jnu.ac.kr (I.P.); doll517@naver.com (I.K.); kochay@jnu.ac.kr (K.-O.C.); 2 Department of Biomedical Sciences, Research Center for Aging and Geriatrics, Research Institute of Medical Sciences, Chonnam National University Medical School, Gwangju 501-190, Korea; 3 COTDE Inc. 19-3, Ugakgol-gil, Susin-myeon, Cheonan-si, Chungcheongnam-do 330-882, Korea; daniel@cotde.co.kr; 4 Department of Obstetrics and Gynecology, Chonnam National University Medical School, Gwangju 501-190, Korea; seokmo2001@yahoo.co.kr; 5 Department of Biochemistry, Dental Science Research Institute, School of Dentistry, Chonnam National University and Korea Mouse Phenotype Center, Gwangju 500-757, Korea; thlee83@jnu.ac.kr

**Keywords:** PTEN, hydrogen peroxide, *tert*-butyl hydroperoxide, Trx system, signaling, tumorigenesis

## Abstract

Organic peroxides and hydroperoxides are skin tumor promoters. Free radical derivatives from these compounds are presumed to be the prominent mediators of tumor promotion. However, the molecular targets of these species are unknown. Phosphatase and tensin homologs deleted on chromosome 10 (PTEN) are tumor suppressors that play important roles in cell growth, proliferation, and cell survival by negative regulation of phosphoinositol-3-kinase/protein kinase B signaling. PTEN is reversibly oxidized in various cells by exogenous and endogenous hydrogen peroxide. Oxidized PTEN is converted back to the reduced form by cellular reducing agents, predominantly by the thioredoxin (Trx) system. Here, the role of *tert*-butyl hydroperoxide (*t*-BHP) in redox regulation of PTEN was analyzed by using cell-based and in vitro assays. Exposure to *t*-BHP led to oxidation of recombinant PTEN. In contrast to H_2_O_2_, PTEN oxidation by *t*-BHP was irreversible in HeLa cells. However, oxidized PTEN was reduced by exogenous Trx system. Taken together, these results indicate that *t*-BHP induces PTEN oxidation and inhibits Trx system, which results in irreversible PTEN oxidation in HeLa cells. Collectively, these results suggest a novel mechanism of *t*-BHP in the promotion of tumorigenesis.

## 1. Introduction

Humans are exposed to various organic peroxides and hydroperoxides contained in cosmetics, chemicals, and foods [1,2]. Peroxides are also generated endogenously during the oxidation of fatty acids and protein [1,3]. A substantial increase in peroxides in vitro and in vivo is generally associated with ubiquitous damage and influences the etiology of diseases, such as aging and cancer [2,4]. Substantial evidence suggests that the tumor promoter activity of peroxides and hydroperoxides involves their ability to generate free radical derivatives [5,6,7]. These radicals participate in substitution, addition or hydrogen-abstraction reactions resulting in protein oxidation or alkylation, lipid peroxidation, and/or DNA damage [8]. *Tert*-Butyl hydroperoxide (*t*-BHP) is an organic hydroperoxide which is broadly used in industry for oxidation reactions. *t*-BHP has been studied as a model to investigate the role of radical intermediates in tumor promotion [9]. In vivo animal studies revealed that *t*-BHP induces the formation of DNA adducts [10] and promotes skin tumors in sensitivity to carcinogenesis (SENCAR) mice [9]. However, molecular targets of *t*-BHP involved in carcinogenesis are poorly understood. 

Phosphatase and tensin homolog deleted on chromosome 10 (PTEN) is a potent tumor suppressor gene that is frequently lost from a region of chromosome 10q23 in a variety of human cancers, including tumors in the endometrium [11], brain [12,13], skin [14], breast [15], and prostate [16]. PTEN is also involved in other human diseases, such as diabetes [17] and autism spectrum disorders [18]. PTEN is considered as the main negative regulator of the phosphoinositol-3-kinase/protein kinase B (PI3K/Akt) signaling pathway, which is an important intracellular signaling pathway in many cellular processes, including cell growth, proliferation, cell survival, transcription, and protein synthesis [19,20,21,22]. PTEN is regulated by transcriptional, post-transcriptional, and post-translational modulations, subcellular localization and binding partners [23]. Hydrogen peroxide (H_2_O_2_) that is produced exogenously and endogenously can oxidize and inactivate PTEN [24,25,26]. H_2_O_2_-oxidized PTEN forms a disulfide bond between cysteine residues 124 (Cys^124^) and 71 (Cys^71^) [27,28], resulting in inactivation of PTEN phosphatase activity. Physiological PTEN oxidation is a reversible process. Oxidized PTEN is reversibly converted back to the reduced form by the intracellular reducing systems, such as thioredoxin (Trx) and glutaredoxin (Grx) systems. Oxidized PTEN is reduced much more effectively by the Trx system than by the Grx or glutathione (GSH) systems in vitro [29]. Depletion of Trx markedly reduces the rate of PTEN reduction in cells [29].

The Trx system consists of Trx, thioredoxin reductase (TrxR) and nicotinamide adenine dinucleotide phosphate (NADPH). It is important to maintain the reduced dithiol status of proteins under physiological and pathological conditions [30]. The well-characterized and ubiquitous Trx systems present in living organisms are the cytosolic thioredoxin reductase (TrxR1) and its classic substrate, Trx1. Trx1 is a general disulfide reductase that acts as an antioxidant by catalyzing the reduction of disulfide bonds in a variety of proteins through both NADPH-dependent and oxidoreductase-dependent systems [31,32].

Studies on redox regulation of PTEN have focused mainly on the H_2_O_2_ in the past decades [24,33,34]. However, PTEN oxidation capability of organic hydroperoxides is poorly described. 15-Hydroperoxy-eicosatetraenoic acid (15-HpETE) has a strong activity to induce the oxidation of protein tyrosine phosphatases (PTP) in vitro as compared to H_2_O_2_ [35]. PTEN, as a member of the PTP superfamily, may share similar responsiveness to other organic hydroperoxides. To prove this hypothesis, we used *t*-BHP, an organic hydroperoxide with tumor-promoting activity. Herein, we showed that *t*-BHP irreversibly oxidized PTEN and inhibited the reduction of oxidized PTEN by the Trx system.

## 2. Results

### 2.1. In Vitro Oxidation of Recombinant PTEN by t-BHP

**Figure 1:**
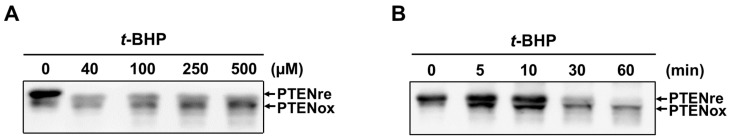
Effects of *t*-BHP on redox state of recombinant PTEN. Purified recombinant PTEN (oxidized form) was pre-reduced with 1 mM dithiothreitol (DTT) for 2 h in degassed assay buffer prior to being applied to NAP-5 desalting column equilibrated with degassed assay buffer to remove excess DTT. Samples were then incubated with the indicated concentration of *t*-BHP for 30 min (**A**), or for increasing periods of time with 0.5 mM *t*-BHP (**B**). After indicated treatment, samples were alkylated with 2 mM NEM. All samples were fractionated by non-reducing SDS-PAGE followed by immunoblot analysis with PTEN antibody. All blot data are representative of at least three separate experiments.

To investigate the effects of *t*-BHP on the redox state of PTEN, pre-reduced recombinant PTEN was incubated with various concentrations of *t*-BHP (0–0.5 mM) for 30 min, or increasing periods of time (0–60 min) with 0.5 mM *t*-BHP. After incubation, the reaction was quenched by *N*-ethylmaleimide (NEM) for 10 min to block free sulfhydryls in order to prevent further reaction. The exposure of recombinant PTEN to *t*-BHP resulted in the increase of faster migrating bands in non-reducing sodium dodecyl sulfate polyacrylamide gel electrophoresis (SDS-PAGE) than in the absence of *t*-BHP (Figure 1A,B). As we have previously demonstrated, the faster migrating bands corresponded to the oxidized PTEN [27]. Purified human PTEN was oxidized by *t*-BHP in concentration- and time-dependent manners.

### 2.2. Irreversible Oxidation of PTEN in HeLa Cells Exposed to t-BHP 

**Figure 2:**
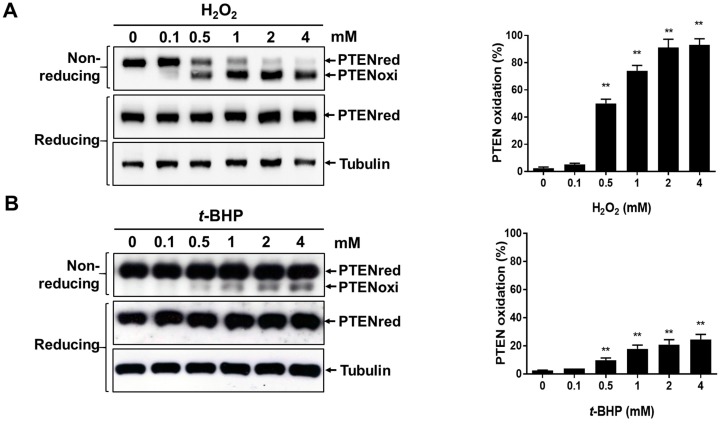

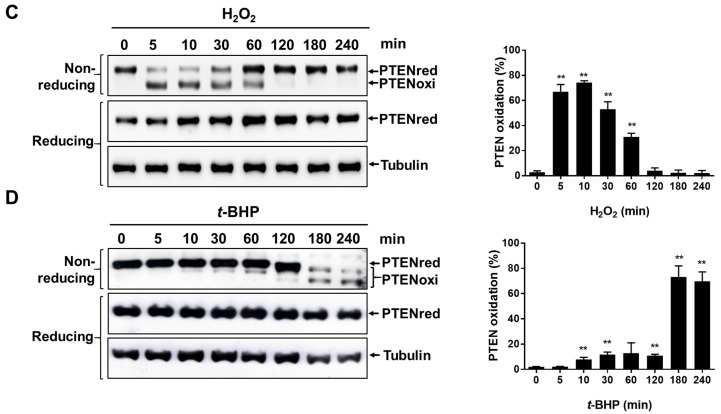
Effects of *t*-BHP and H_2_O_2_ on redox state of PTEN in HeLa cells. Hela cells were incubated either with the indicated concentrations of H_2_O_2_ (**A**) or *t*-BHP (**B**) for 30 min or incubated for the indicated times either with 1 mM H_2_O_2_ (**C**) or 1 mM t-BHP (**D**). After treatment with 1 mg/mL of catalase for 5 min, cellular protein extracts were then alkylated with 20 mM NEM and subjected to non-reducing or reducing SDS-PAGE followed by Western blot analysis with antibodies to PTEN. Tubulin levels were used as a loading control. All blot data are representative of at least three separate experiments. The intensity of PTEN bands were quantitated with ImageJ software (ImageJ 1.50i, National institutes of Health, Bethesda, MD, USA), ** *p* < 0.05 as compared to control.

Next, we investigated whether cellular PTEN is also oxidized in the cells when exposed to *t*-BHP. HeLa cells were incubated with various concentrations of H_2_O_2_ or *t*-BHP for 30 min, or with 1 mM H_2_O_2_ or *t*-BHP for various times. Cell extracts were exposed to NEM to terminate the reaction and then subjected to non-reducing or reducing electrophoresis, followed by immunoblot analysis with antibodies to PTEN. Exposure of cells to 0.1 mM H_2_O_2_ resulted in PTEN oxidation, and the band intensity of oxidized PTEN increased as H_2_O_2_ concentration increased in the non-reducing condition (Figure 2A). Similarly, exposure of cells to 0.5 mM *t*-BHP resulted in PTEN oxidation, and this band intensity also increased as *t*-BHP concentration increases in non-reducing condition (Figure 2B). Oxidized PTEN finally reverted to the slower migrating form as incubation was continued after treatment with 1 mM H_2_O_2_ in HeLa cells (Figure 2C, non-reducing condition), as has been described previously [25], suggesting that PTEN that is oxidized and inactivated by H_2_O_2_ is eventually reduced by the cellular redox system. In contrast, the oxidized PTEN observed in HeLa cells exposed to 1 mM *t*-BHP was not reduced when incubation was extended, as occurred with H_2_O_2_ treatment (Figure 2D, non-reducing condition). When extracts derived from HeLa cells exposed to H_2_O_2_ or *t*-BHP were incubated with 2-mercaptoethanol before gel electrophoresis, only a single band of PTEN was detected (Figure 2A,D reducing condition), indicating that the protein in the higher mobility form under the non-reducing condition contained a disulfide bond.

### 2.3. Exogenous Trx System Reduces Cellular PTEN Oxidized by t-BHP

The reduction of H_2_O_2_-oxidized PTEN was predominantly mediated by Trx system in cells, as described previously [34]. The irreversible oxidation of PTEN by organic hydroperoxide *t*-BHP likely reflects the inhibition of the Trx system.

**Figure 3:**
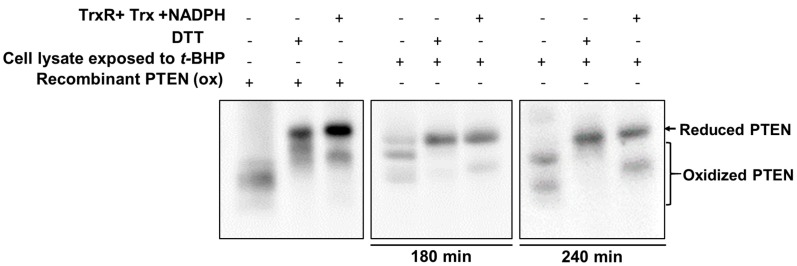
Reduction of the oxidized PTEN by exogenous Trx system. HeLa cells were treated with 0.5 mM *t*-BHP to oxidize endogenous PTEN for 180 and 240 min. After treatment, samples were alkylated with 20 mM NEM in the presence of 20 mM Tris-HCl (pH 7.4), 150 mM NaCl, 5% glycerol, 0.1% Nonidet P40 and protease inhibitor, the supernatant was applied to NAP-5 desalting column equilibrated with lysis buffer to remove excess NEM. Recombinant PTEN (oxidized form) and cleared cell lysates were untreated or treated with DTT or with a combination of thioredoxin (Trx) system components (Trx1, TrxR1 and NADPH) and analyzed on non-reducing SDS-PAGE, followed by immunoblotting with PTEN antibodies.

To test whether *t*-BHP inhibits Trx system activity, the effect of the exogenous Trx system on PTEN reduction was analyzed. To induce oxidation of PTEN, cultured HeLa cells were initially treated with 0.5 mM *t*-BHP for 120 or 180 min. After treatment, samples were alkylated with 20 mM NEM in the presence of 20 mM Tris-HCl, pH 7.4, 150 mM NaCl, 5% glycerol, 0.1% Nonidet P40, and protease inhibitor. The supernatant was applied to a NAP-5 desalting column equilibrated with lysis buffer to remove excess NEM. Recombinant PTEN (oxidized form) and cleared cell lysates were incubated with the combination of the cytosolic Trx system components (Trx1, TrxR1, and NADPH) or with the strong artificial reducing agent DTT. The oxidized recombinant PTEN was reduced by both DTT and the exogenous Trx redox system (Figure 3, left panel). Cellular PTEN oxidized by *t*-BHP was also reduced by DTT (Figure 3, middle panel) and by the exogenous Trx redox system (Figure 3, right panel). These results provide indirect evidence that impairment of the reduction process of *t*-BHP-oxidized PTEN is mediated by the inhibition of the Trx system.

### 2.4. t-BHP Inhibits the Reduction of Oxidized PTEN by the Trx System

**Figure 4:**
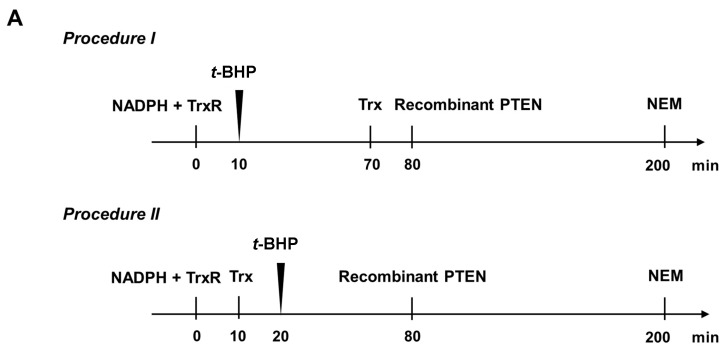

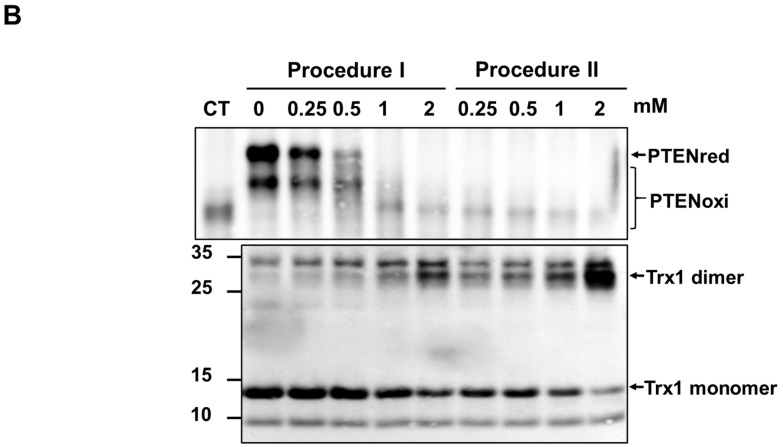
Effects of *t*-BHP on the reduction of oxidized PTEN by the Trx system. The experimental procedures are shown (**A**). PTEN and Trx1 were analyzed by non-reducing SDS-PAGE followed by immunoblotting (**B**). CT represents recombinant PTEN without any treatment.

To further characterize whether *t*-BHP can irreversibly oxidize PTEN by blocking TrxR/Trx/NADPH system, the Trx system components were incubated with various concentrations of *t*-BHP and then incubated with recombinant PTEN. The experiments were summarized in Figure 4A. As a first experimental setup, TrxR1 was pre-reduced with NADPH for 10 min. To test whether *t*-BHP affects Trx system activity, various concentrations of *t*-BHP was treated to the reaction mixture, before Trx1 addition (Figure 4A, procedure I). Reduction of PTEN was decreased in a *t*-BHP concentration-dependent manner (Figure 4B, procedure I). Next, *t*-BHP was treated to the reaction mixture after Trx1 addition (Figure 4A, procedure II). The inhibitory activity of *t*-BHP on PTEN reduction by Trx system was more efficient in procedure II as compared to procedure I. Interestingly, although the formation of dimeric Trx1 increased as the *t*-BHP increases in procedures I and II, the amount of the monomeric form of Trx1 in procedure II was much less than the amount in procedure I (Figure 4B, lower panel). Given that Trx1 is functional as a monomer in redox reactions [36], these data suggest that *t*-BHP inhibits the reduction of oxidized PTEN via targeting the Trx system. 

## 3. Discussion

The molecular mechanisms of organic peroxides and hydroperoxides involved in tumor promotion are central in the study of free radicals. *t*-BHP is a common organic hydroperoxide which has cytotoxicity and tumor-promoting activity. These properties are mediated by its metabolites. *t*-BHP is extensively metabolized in the target issues to form several free radical intermediates, including phenoxyl, peroxyl, alkoxyl, and alkyl radical derivatives in murine keratinocytes [9], and hemoglobin-thiyl and methyl radicals in rat liver and stomach [10]. The cytotoxic effects of *t*-BHP are involved in glutathione depletion [37], hemoglobin oxidative denaturation, hemolysis, and erythrocyte membrane lipid peroxidation [38,39], inner mitochondrial membrane permeabilization [40], DNA single-strand breakage [41,42], and apoptosis [43]. However, the specific in vivo targets are poorly understood. 

PI3K is activated by various cellular stimuli [44,45], resulting in the phosphorylation of phosphatidylinositol (4,5)-bisphosphate (PIP2) to phosphatidylinositol (3,4,5)-trisphosphate (PIP3). In the cytoplasm, PTEN dephosphorylates PIP3 to PIP2. The PI3K/Akt signaling pathway is negatively regulated by PTEN, which inhibits cell growth, proliferation, cell survival, protein synthesis, and transcription. PTEN thus plays a critical role for the inhibition of carcinogenesis. The present study shows for the first time that the PTEN tumor suppressor is one of the target molecules of *t*-BHP.

We have previously reported that exposure to H_2_O_2_ leads to PTEN oxidation harboring reversible intramolecular disulfide bonds between Cys^124^ and Cys^71^ [27]. The cysteine residues can be further oxidized into irreversible sulphinic (–SO_2_H) or sulphonic (–SO_3_H) acids upon ongoing exposure to oxidants, which cannot be reduced by cellular redox systems or artificial reductants. Presently, treatment of cells with *t*-BHP resulted in PTEN oxidation that could not revert (Figure 2B,D non-reducing condition), while PTEN oxidized by H_2_O_2_ was reverted to a reduced form during the incubation after the treatment. However, this effect was completely reversed by treatment with 2-mercaptoethanol or DTT (Figure 2B,D, reducing condition, and Figure 3, with DTT treatment), suggesting that *t*-BHP-mediated oxidation of PTEN proceeds through a mechanism other than the formation of sulphinic or sulphonic acids.

Similar to treatment with H_2_O_2_, the oxidized PTEN band that corresponds to H_2_O_2_-oxidized PTEN was observed until 60 min of incubation upon treatment with *t*-BHP. However, prolonged incubation with *t*-BHP over 120 min resulted in the formation of two PTEN bands that are distinct from the bands observed after exposure to H_2_O_2_. None of these two bands corresponded to the reduced PTEN or oxidized PTEN with single disulfide bridge. Therefore, we speculate that these two bands may represent a differentially oxidized form of PTEN other than a single disulfide bridge. Based on our previous reports, one candidate for the upper band observed at 120 min might be glutathionylated PTEN [46], an intermediate form for PTEN reduction by glutathione. The lower band might represent the hyperoxidized PTEN that contains additional disulfide bonds, as was observed in the oxidation of Trx1 [47]. To confirm this hypothesis, further validation is needed. The total amount of PTEN was decreased at 180 and 240 min of incubation with *t*-BHP in HeLa cells. E3 ubiquitin ligase is known to regulate the PTEN protein level via polyubiquitination and proteasome-mediated degradation [48]. The decrease of *t*-BHP-oxidized PTEN could be associated with polyubiquitination and proteasome-mediated degradation. This speculation needs further investigation.

**Figure 5:**
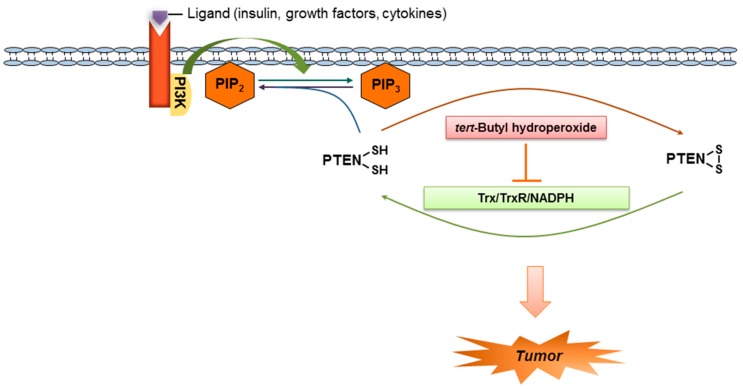
A schematic model for the redox regulation of the tumor suppressor PTEN by the thioredoxin system and the tumor promoter *t*-BHP. PIP3K: phosphoinositol-3-kinase; PIP2: phosphatidylinositol (4,5)-bisphosphate; PIP3: phosphatidylinositol (3,4,5)-trisphosphate.

Oxidized PTEN is gradually converted back to the reduced form by cellular reducing agents, predominantly by the Trx system. Trx is a small protein present in all living cells that reduces disulfide bonds or oxidizes sulfhydryls in proteins using a conserved sequence (Trp-Cys-Pro-Cys). The active site of Trx is blocked when dimerized [36]. The effect of *t*-BHP on redox state of PTEN in cells implicates the blockage of Trx system activity, which was clearly demonstrated by the observation that *t*-BHP mediated oxidation of cellular PTEN was reduced by the exogenous Trx system (Figure 3). Furthermore, treatment with *t*-BHP induced Trx dimer formation, which resulted in the impairment of reduction of oxidized PTEN (Figure 4). Taken together, the present findings uncover a previously unrecognized *t*-BHP-induced tumor promotion pathway (Figure 5). *t*-BHP induces irreversible PTEN oxidation by direct oxidation of PTEN and by blocking Trx system, a critical PTEN reduction system, which results in the loss of PTEN tumor suppressor activity and the activation of the PI3K/Akt signaling pathway that leads to carcinogenesis.

Our results provide new lines of evidence into the role of PTEN in organic hydroperoxides-mediated tumor promotion. Further studies are required to more conclusively explore the correlation between organic hydroperoxides and PTEN in vivo.

## 4. Materials and Methods

### 4.1. Materials and Reagents

The recombinant wild type PTEN and Trx1 were purified as previously described [27]. Thioredoxin reductase was purified from the mouse liver as described previously [49]. β-Nicotinamide adenine dinucleotide 2′-phosphate reduced tetrasodium salt hydrate (NADPH), *N*-ethylmaleimide (NEM), *N*,*N*-dimethylformamide (DMF), and dl-dithiothreitol (dl-DTT) were purchased from Sigma-Aldrich (St. Louis, MO, USA). Hydrogen peroxide (H_2_O_2_) was purchased from OCI Company Ltd. (Seoul, Korea). *tert*-Butyl hydroperoxide (*t*-BHP) was purchased from Alfa Aesar (Ward Hill, MA, USA). Protease inhibitor (Complete ULTRA Tablets) was purchased from Roche Diagnostics GmbH (Indianapolis, IN, USA). PTEN antibody was prepared as previously described [50]. Anti-Trx1 and anti-rabbit IgG horseradish peroxidase-conjugated antibodies were purchased from Ab Frontier (Daejeon, Korea). Anti-tubulin antibodies were from Sigma-Aldrich. Immobilon Western chemiluminescence horseradish peroxidase substrate was purchased from Millipore Corporation (Billerica, MA, USA). NAP-5 Columns (Sephadex G-25 DNA Grade) were purchased from GE Healthcare (Little Chalfont, UK). Dulbecco’s modified Eagle’s medium (DMEM) and fetal bovine serum (FBS) were purchased from Capricorn Scientific GmbH (Ebsdorfergrund, Germany).

### 4.2. Cell Culture and Oxidation of Cellular PTEN

HeLa cells were cultured in DMEM containing 10% FBS and maintained in a humidified 5% CO_2_ incubator at 37 °C. To begin an experiment, an 80% confluent monolayer of HeLa cells was rinsed three times with phosphate-buffered saline (PBS) and incubated in FBS-free DMEM at 37 °C for 30 min with the indicated concentrations of H_2_O_2_ or *t*-BHP, or for the indicated times after treatment with 1 mM H_2_O_2_ or *t*-BHP in FBS-free DMEM. Cells were treated with 1 mg/ml catalase for 5 min. Cellular protein extracts were alkylated with 20 mM NEM in the presence of 20 mM Tris-HCl (pH 7.4), 150 mM NaCl, 5% glycerol, 0.1% Nonidet P40 and protease inhibitor. NEM was used to irreversibly block all unoxidized thiol groups in intact cells. Lysates were sonicated and centrifuged at 13,200 rpm for 10 min at 4 °C. Cleared lysates were mixed with reducing electrophoresis gel-loading buffer (60 mM Tris, pH 6.8, 25% glycerol, 2% SDS, 5% 2-mercaptoethanol, and 0.5% bromophenol) or non-reducing sample buffer without reducing agents and then subjected to 8% SDS-PAGE. Proteins were transferred to NC membrane (GE Healthcare Life Sciences) and blocked with 5% skim milk in Tris-buffered saline-Tween 20 (TBST), followed by incubation with primary antibodies to PTEN, Trx1 or tubulin, and then with secondary antibodies (BD Transduction Laboratories, San Jose, CA, USA). Immune complexes were visualized by chemiluminescence using ECL solution (Thermo Scientific, Waltham, MA, USA).

### 4.3. Oxidation of Purified PTEN

Purified PTEN was pre-reduced with 1 mM DTT for 2 h and passed through NAP-5 column which is pre-equilibrated with PTEN assay buffer (100 mM Tris-HCl (pH 8.0), 2 mM EDTA, 0.1% BSA), to remove DTT before starting the assay. The PTEN assay buffer was deoxygenated using a stream of argon for a minimum 30 min before use. The pre-reduced PTEN was exposed to 0.5 mM *t*-BHP for indicated times or to various concentrations for 30 min at room temperature. Then the reactions were stopped by 2 mM NEM. Samples were subjected to non-reducing SDS-PAGE and immunoblotting for PTEN.

### 4.4. Treatment of Cell Lysates with Trx System Components

HeLa cells were treated with 0.5 mM *t*-BHP for 120 or 180 min. After treatment, samples were alkylated with 20 mM NEM in the presence of 20 mM Tris-HCl (pH 7.4), 150 mM NaCl, 5% glycerol, 0.1% Nonidet P40 and protease inhibitors. The supernatant was applied to NAP-5 desalting column equilibrated with lysis buffer to remove excess NEM. The cleared cell lysates were treated with a combination of Trx system components (100 µM NADPH, purified TrxR1 and 2 µg of purified Trx1) or 1 mM DTT for 2 h. Before the reaction with cell lysate, TrxR1 and Trx1 were pre-incubated with NADPH for 10 min.

### 4.5. Treatment of t-BHP on the Trx System

Purified TrxR1 were pre-incubated with 100 µM NADPH for 10 min and then mixed with 2 µg purified Trx1 to obtain the active form of Trx1. The resulting reaction solution was then incubated with 2 µg of purified recombinant PTEN (oxidized form) for 120 min and the reaction was stopped by 2 mM NEM. To test the effect of *t*-BHP on PTEN reduction by Trx system, various concentrations of *t*-BHP were treated to the reaction mixture before (procedure I) or after (procedure II) Trx1 addition. All steps were carried out at room temperature. PTEN and Trx1 were detected by non-reducing SDS-PAGE followed by immunoblotting.

### 4.6. Statistical Analysis

All values are expressed as means standard deviation (SD). Statistical analysis was performed with Student’s *t*-tests. *p*-values < 0.05 were considered statistically significant.

## 5. Conclusions

This study showed PTEN was irreversibly oxidized in cells when exposed to *t*-BHP, in contrast to H_2_O_2_. Oxidized PTEN by *t*-BHP in HeLa cells was reduced by the exogenous Trx system. Addition of *t*-BHP to the exogenous Trx system inhibited the reduction of oxidized PTEN and induced the formation of an inactive Trx1 dimer. These results demonstrate that the organic hydroperoxide *t*-BHP might promote tumorigenesis via the irreversible oxidation of the tumor suppressor PTEN in cells.
